# A healthful plant-based diet is associated with higher health-related quality of life among older adults independent of circulating CRP: a cross-sectional analysis from the Lifelines Cohort Study

**DOI:** 10.1017/jns.2025.10023

**Published:** 2025-08-04

**Authors:** Kerstin Schorr, Marian Beekman, Venetka Agayn, Jeanne H.M. de Vries, Lisette C.P.G.M. de Groot, P. Eline Slagboom

**Affiliations:** 1 Section of Molecular Epidemiology, Department of Biomedical Data Sciences, Leiden University Medical Center, Einthovenweg 20, 2333 ZC Leiden, The Netherlands; 2 Innoso BV, Ruychrocklaan 68, 2597 EP The Hague, The Netherlands; 3 Division of Human Nutrition and Health, Wageningen University, P.O. Box 176700 AA Wageningen, The Netherlands

**Keywords:** Dietary pattern, Inflammation, Older adults, Plant-based diet, Subjective well-being, FFQ, food frequency questionnaire, hPDI, healthful plant-based diet index, hsCRP, high-sensitivity C-reactive protein, MCS, mental component score, PDI, Plant-based diet index, PCS, physical component score, uPDI, unhealthful plant-based diet index

## Abstract

Plant-based diets (PBD) have been found to be environmentally sustainable and beneficial for health. Observational research showed that higher plant-based diet quality improves health-related quality of life (HRQoL) in adult women, however this is unclear for older adults. This association may be due to anti-inflammatory properties of PBD. Older adults, prone to chronic inflammation, may therefore profit from PBD. We investigated the relation between PBD and HRQoL in older adults of both sexes and tested whether the effects are associated with circulating high-sensitivity C-reactive protein (hsCRP) levels. We used data of the population-based Lifelines Cohort Study (n = 6,635, mean age = 65.2 years) and a subsample in which hsCRP was measured (n = 2,251, mean age = 65.2 years). We applied a plant-based diet index measuring adherence to a healthful (hPDI) and an unhealthful (uPDI) plant-based diet based on food frequency questionnaires. The RAND-36 questionnaire was applied as measure of HRQoL, from which we derived physical and mental HRQoL. Older adults with the highest adherence to a hPDI had respectively 15% and 12% greater odds for high physical quality of life and mental quality of life. Meanwhile, higher adherence to uPDI was associated with respectively 16% and 13% lower odds for high physical and mental quality of life. An additive but no interactive effect of hsCRP on the association between PBD and HRQoL has been observed. Adherence to a healthful plant-based diet and circulating levels of inflammation are independently associated with physical and mental HRQoL. Mechanisms other than inflammation through which PBD could influence HRQoL may be explored in further research.

## Introduction

Plant-based diets are becoming increasingly popular, and dietary organizations recommend a shift towards a more plant-based diet^(1–3)^. The growing interest may partly be due to the fact that a plant-based diet contributes less to greenhouse gas emissions and thus to climate change^(3)^. Apart from that, many health benefits of healthful plant-based diets have been discussed, primarily a reduced risk for age-related diseases, such as cardiovascular and metabolic diseases, cognitive impairment, and health-related quality of life (hrQoL)^(4–8)^. With advancing age chronic illnesses and multi-morbidity become more prevalent. This highlights the benefits of adopting a plant-based diet for quality of life and mental and physical quality of life beyond the effects on health. Generally, plant-based diets are considered to be low in animal products but not strictly vegan. Therefore, PBD can be a suitable dietary pattern for older adults helping to avoid low protein intake and concomitant muscle loss and thereby contributing to better physical quality of life.

Health-related quality of life (hrQoL) has been defined as “how well a person functions in their life and his or her perceived wellbeing in physical, mental, and social domains of health”^(9)^. HrQoL and self-rated health have been associated with various different lifestyle or sociodemographic factors in older adults. Female sex, higher age, physical inactivity, and the prevalence of chronic conditions have been associated with poor hrQoL^(10,11)^. In contrast, a healthful plant-based diet showed a positive effect on hrQoL middle-aged healthy women^(7)^. There could however also be potential drawbacks in following plant-based diets. Recently it has been suggested that plant-based diet quality may be related to depressive symptoms. Particularly an unhealthful plant-based diet has been found to be associated with an increased risk for depression^(12,13)^. This suggests not only a potential link between dietary intake and hrQoL but also highlights the importance of diet quality.

An added benefit of plant-based diets may lie in their anti-inflammatory properties^(14–16)^. Chronic inflammation, in the absence of infection, is prevalent especially among older adults. This so called inflammaging is part of the ageing process and may negatively affect hrQoL in older ages, as elevated levels of inflammatory markers may potentially be linked to decreased quality of life and well-being^(17–19)^. It has been hypothesized that the association between a healthful plant-based diet and higher quality of life may be due to an anti-inflammatory effect of a plant-based diet^(7)^. Despite this hypothesis, a moderating effect of circulating inflammatory markers on quality of life in older adults has not been investigated yet. Current literature, however, has focused mainly on the effects of plant-based diets in younger adults, usually devoid of chronic inflammation, and mainly in women^(20)^. Therefore, it needs to be elucidated whether the effect of adherence to healthful plant-based diets on mental and physical quality of life both in older men and women can be explained by chronic inflammation.

Here we investigate the relation between adherence to a plant-based diet and wellbeing in men and women above 60 years and whether such associations are independent of chronic inflammation established by circulating hsCRP. We will assess 1) how adherence to a healthful and unhealthful plant-based diet is associated with physical and mental quality of life in adults of different age groups and sexes and 2) whether this relationship is dependent on circulating CRP levels as proxy of inflammation levels.

## Material and methods

### Study population

The current study investigated data from the Lifelines Cohort Study (https://www.lifelines-biobank.com/)^(21)^. The Lifelines Cohort study is a multi-disciplinary prospective population-based cohort study examining the health and health-related behaviors of 167,729 persons living in the North of the Netherlands in a unique three-generation design. It employs a broad range of investigative procedures in assessing the biomedical, socio-demographic, behavioral, physical, and psychological factors which contribute to the health and disease of the general population, with a special focus on multi-morbidity and complex genetics. The LifeLines Cohort Study is conducted according to the principles of the Declaration of Helsinki and is approved by the medical ethical committee of the University Medical Center Groningen, The Netherlands^(21)^. Baseline assessments were carried out from 2007 to 2013, followed by a second (2014–2017) and third (2019–2023) assessment round, interspersed with follow-up questionnaires.

For the purpose of this study, we included participants, from whom baseline food frequency questionnaire (FFQ) data, as well as quality of life data (assessed by RAND-36 questionnaire) was available. After excluding participants with unreliable energy intake (<800 kcal and >4200 kcal for men and <500 kcal and >3500 kcal for women) or missing data (n = 125,244) were excluded, 41,975 participants remained, among which n = 6,528 were 60 years or above (Table 1) and n = 35,447 between 18–59 years (Supplementary Table 2). The association of circulating high sensitivity C-reactive protein levels with hrQoL was analyzed in a subgroup of the sample with hsCRP data available (n_older adults_ = 2,198, n_younger adults_ = 14,524).

**Table 1.** Baseline characteristics of lifelines participants in younger (<60 years) and older adults (≥60 years)

	Younger (n = 35,447)		Older (n = 6,528)	
	Mean	SD	Mean	SD
hPDI [18–90]	53.6	7.2	56.5	6.8
uPDI [18–90]	54.5	7.1	50.4	6.2
n	35,447		6,528	
Females (n%)	21,464		3,405	
Age (mean±SD)	42.5	9.9	65.1	4.8
BMI [kg/m^2^]	25.4	3.9	26.5	3.6
Physical Activity [h/week]	8.2	10.7	6.7	7.5
Nr of age-related diseases [0–7]	0.5	0.7	0.9	0.9
Monthly income				
(<1500€)	5052		1036	
(1500–3000€)	16795		3737	
(>3000€)	13600		1755	
Current smoker	6082		536	
Depression diagnosed	3396		508	
PCS (mean)	53.7	6.9	51.7	7.3
MCS (mean)	50.8	8.2	53.4	7.1
hsCRP	2.29	4.00	2.44	4.60

hPDI = healthful plant-based diet index, uPDI = unhealthful plant-based diet index, BMI = body mass index, Physical CS = physical component score, Mental CS = mental component score, hsCRP = high-sensitivity C-reactive protein.

### Plant-based diet index

We applied a plant-based diet index to quantify the adherence to a plant-based diet and quality of foods consumed. The plant-based diet index (PDI) was adapted from previous publications and calculated from FFQ data^(22)^. The FFQ in the Lifelines Cohort Study was implemented in the form of a flower FFQ, meaning it was split into smaller questionnaires and spread between assessments^(23–25)^. FFQ items were classified into food groups, as displayed (Supplementary Table 1). Categorization of food items was based on previous publications and recommendations of the Netherlands nutrition center (Voedingcentrum^1^). In contrast to the original index^(22)^, intake of certain margarines was included in the vegetable oil category, in accordance with current recommendations on healthy fats.

The intake in each food group (in g/d) was then divided into cohort-specific quintiles and points awarded per quintile. For the healthful PDI (hPDI) scale healthful plant-based food groups were scored positively (1 = lowest quintile, 5 = highest quintile), while less healthy food groups were reverse-scaled (1 = highest quintile, 5 = lowest quintile). This procedure was reversed for the unhealthful PDI (uPDI), so that healthy plant foods were scored reversely, while less healthy plant foods were scored positively. Animal food groups were always scored reversely, with the lowest quintile receiving 5 points and the highest quintile 1 point. This results in indices ranging from 18–90, with higher index scores indicate higher adherence to the respective dietary pattern. As such, the score allows for comparison of plant-based diets of varying composition. To account for differences in energy intake, the indices were adjusted for overall energy-intake using the residual method, by regressing energy intake on intake per food group^(26)^.

### Quality of life

Quality of life was measured by a Dutch translated version of the RAND-36 questionnaire, a tool to assess quality of life in eight subdomains, covering areas of both physical and mental health^(27)^. Subdomains consist of physical functioning, bodily pain, role limitations due to physical health problems, role limitations due to personal or emotional problems, emotional well-being, social functioning, energy/fatigue, and general health perceptions. Subdomain scores were derived according to scoring instructions^2^. From the scores of the subdomains, physical and mental components scores (PCS and MCS) were calculated by standardizing the scores using population-specific factors for the Dutch population. In a second step, to calculate the aggregated PCS and MCS, the standardized subdomain scales are multiplied by their respective factor coefficient and summed. In a last step, the component scores are normalized, resulting in a score in which a mean of 50 (and standard deviation of 10) represents the mean of the Dutch population. The computation of the component scores and population-specific factors are described in detail elsewhere^(28,29)^. For the purpose of this study, the component scores were binarized by the median, in order to allow for comparison of subjects with low and high quality of life.

### Inflammatory marker

Blood samples were collected via venipuncture before 10 am in the morning in fasted participants between 2008 and 2013. The blood samples were placed at 4°C and transported from the LifeLines research site to the LifeLines laboratory in Groningen, under tightly controlled and continuously monitored conditions. From the LifeLines laboratory, the samples were directly transferred to the central laboratory of the University Medical Center Groningen, to perform routine clinical chemistry assays on fresh samples^(21)^. High-sensitivity CRP was measured in heparin plasma using kits from two different suppliers, either by CardioPhase hsCRP, Siemens Healthcare Diagnostics, Marburg, Germany or CRPL3, Roche Diagnostics, Mannheim, Germany. Measurements were carried out at the Groningen University Medical Center^(21)^.

### Covariates

Age, sex, body mass index (BMI), physical activity, income, disease prevalence, smoking, alcohol intake, prevalence of depression and energy intake were considered as covariates. Information on age, sex, BMI, physical activity, income, diagnosed diseases, including depression, smoking behavior and alcohol intake was derived from questionnaires^(21)^. BMI, age, physical activity, nr of diagnosed diseases and alcohol and energy intake were treated as continuous covariates, while smoking, income and depression were categorical. To adjust for disease prevalence, we created a continuous compound variable indicating how many of common age-related diseases (myocardial infarction, hypertension, stroke, cancer, diabetes, and arthritis) were diagnosed in a subject. Physical activity was measured by the Squash questionnaire^(30)^ and reported continuously in minutes/week of moderate and vigorous physical activity^(31)^. To obtain a more interpretable unit we converted this physical activity variable into hours/week. Information on alcohol intake was derived from the FFQ and is reported continuously in g/d. Since a slight increase in total energy intake across tertiles of hPDI was observed, we additionally adjusted for energy intake in kcal/d. Depression was treated as a binary variable (yes/no). Income has also been found to be associated with quality of life in previous research^(32)^. Net income was categorized as low (<1500€), medium (1500€ to 3000€) and high (>3000€/month). Smoking status was reported as never, former or current smoker.

### Statistical analysis

To assess the association between the physical and mental component scores and the plant-based diet indices, a logistic regression with binarized component scores as the outcome was applied. Cut-offs for PCS and MCS in the whole sample were 55.4 and 53.3 respectively. The PDIs were treated as predictor variables and divided into tertiles, while adjusting for age, sex, BMI, physical activity, income, use of prescription medication and energy intake. To investigate differences in the association of PDIs and hrQoL in men and women or different age groups, we test interactions of PDIs with age and sex. To assess the significance of the interaction, we ran an analysis of variance comparing the models with and without interaction term. In case of significant interaction, we stratify by sex/age group (≥60 years and <60 years). Secondly, to assess a potential interaction of circulating hsCRP with PDIs, we add an interaction term to age-stratified models. A potential association between hsCRP and plant-based diet adherence was assessed via linear regression, with hsCRP as outcome and PDIs as predictor variables. As hsCRP is not normally distributed we apply a log-transformation. All models were adjusted for covariates. In the last step, hsCRP was added to the models of the first step (Model 2, Supplementary Material 1). As sensitivity analysis, the association between an overall plant-based diet index and quality of life was assessed using the logistic regression model from the first step (Model 3, Supplementary Material 1). Further, we replicated our findings by applying a linear regression with rank-inverse normal transformed PCS and MCS as the outcome. All analyses were carried out in R.

## Results

### Sample characteristics

Among older adults, above 60 years, those with a high adherence to a healthful plant-based diet (hPDI), i.e., those in the highest tertile of hPDI were more likely to be female, had a lower BMI and were slightly more physically active than those in the lowest tertile of hPDI. Further, those with the highest adherence to hPDI were less likely to take prescription medication and to have a low income. Meanwhile those with a high adherence to an unhealthful plant-based diet, i.e., in the highest tertile of uPDI were more likely male, less physically active and had a lower income as compared to those in the lowest tertile of uPDI (Table 1). Similar differences across tertiles were observed among younger adults (Supplementary Table 2). Among younger adults, those in the highest tertile of hPDI had higher intakes of protein, particularly plant protein, fiber and unsaturated fatty acids compared to those in the highest tertile of uPDI (Supplementary Table 3). HsCRP values were higher among older adults (2.4±4.6) compared to younger adults (2.3±4.0). although the difference was not significant (p = 0.145).

### Plant-based diet index associated with HRQOL

In the whole sample, high adherence to a healthful plant-based diet was associated with higher odds for good physical (OR = 1.15, 95% CI [1.09; 1.20]) and mental quality of life (OR = 1.12 95% CI [1.06; 1.17]). An unhealthful plant-based diet was associated with lower odds for good physical (OR = 0.84 95% CI [0.80; 0.89]) and mental quality of life (OR = 0.87, 95% CI [0.83; 0.92]).

To further answer the question whether these associations are dependent on sex and age, we include interaction terms. No significant interaction was observed between PDIs and sex. For the interaction of PDIs and age, we observe a significant interaction for hPDI*age on PCS (p = 0.021) and uPDI*age on MCS (p<0.001).

**Fig. 1:**
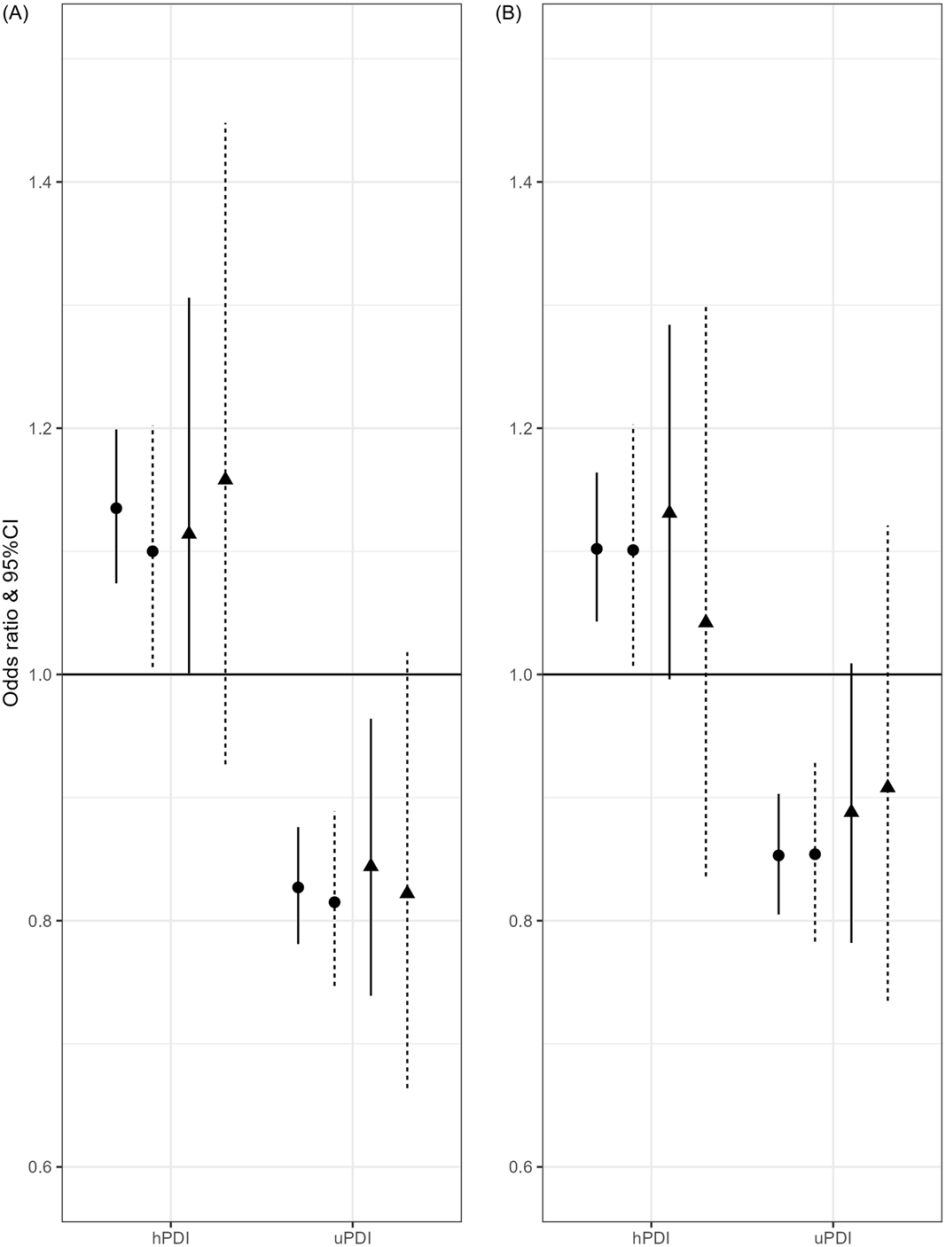
Associations between the highest tertile of healthful (hPDI) and unhealthful (uPDI) plant-based diet and health-related quality of life. A: association with physical component score, B: association with mental component score, ●adults ≥60, ▲adults <60 years, │model without hsCRP, ¦ model with hsCRP.

Subsequently, to further investigate the observed interaction, we stratify our sample with a cut-off of 60 years. In both, younger and older adults, we observe the same direction of effect. Namely, a greater healthful plant-based diet is associated with higher odds for good physical quality of life in younger (OR = 1.14, 95% CI [1.07; 1.20]) and older adults (OR = 1.14, 95% CI [1.00; 1.31]). An unhealthful plant-based diet was associated with lower odds for high mental quality of life in those below (OR = 0.85, 95% CI [0.81; 0.90]) and above 60 years (OR = 0.89, 95% CI [0.78; 1.01]) (Fig. 1), although the association was only borderline significant in older adults. Effect sizes are similar in both age groups, although we observe a loss of significance in the older age group (Table 2).

**Table 2.** Associations between a healthful (hPDI) and unhealthful (uPDI) plant-based diet index with physical (PCS) and mental (MCS) component scores among older (*≥*60 years) adults and younger adults (<60 years)

Diet index	Tertile	Outcome	Age group	OR	95% CI	p-value
hPDI	Medium	PCS	Younger	1.121	1.063; 1.181	<0.001
	High		Younger	1.135	1.074; 1.199	<0.001
	Medium		Older	1.081	0.954; 1.226	0.221
	High		Older	1.144	1.001; 1.306	0.048
	Medium	MCS	Younger	1.056	1.003; 1.113	0.040
	High		Younger	1.102	1.043; 1.164	<0.001
	Medium		Older	1.026	0.911; 1.156	0.673
	High		Older	1.131	0.996; 1.284	0.058
uPDI	Medium	PCS	Younger	0.921	0.874; 0.970	0.002
	High		Younger	0.827	0.781; 0.876	<0.001
	Medium		Older	0.900	0.793; 1.020	0.098
	High		Older	0.844	0.739; 0.964	0.013
	Medium	MCS	Younger	0.900	0.854; 0.948	<0.001
	High		Younger	0.853	0.805; 0.903	<0.001
	Medium		Older	0.923	0.818; 1.040	0.189
	High		Older	0.888	0.782; 1.009	0.068

n_older_=6528, n_younger_=35.447 adjusted for age, sex, BMI, physical activity, medication, income, disease prevalence, smoking, alcohol intake, depression, and energy intake.

hPDI=healthful plant-based diet index, uPDI=unhealthful plant-based diet index, lowest tertile of PDIs is reference category, PCS=physical component score, MCS=mental component score.

### Assessing interactive effect of hsCRP on association between plant-based diets and HRQOL

In order to test if the association between PDIs and hrQoL is explained by circulating hsCRP levels, we add hsCRP to our models. We test if models with an interaction term explains more variation than models testing independent effects of PDIs and hsCRP via anova. No interaction of hPDI or uPDI with hsCRP was observed in any age group. We do observe a significant association of circulating hsCRP with the physical component score in all age groups. Meanwhile, hsCRP was not associated with mental component score in any age group. In younger adults, significant associations of both, high hPDI and uPDI, to physical and mental component scores persist. Among older adults, the associations of diet scores and physical and mental component scores are rendered insignificant. However, effect sizes remain largely comparable, suggesting the loss of significance may be due to lower sample size among the older age group (Table 3, Fig. 1).

**Table 3.** Association of healthful and unhealthful plant-based diet index and circulating hsCRP with physical and mental QoL in younger (<60 years) and older adults (≥60 years)

					Younger adults				Older adults			
	OR [95% CI] Medium Diet	p-value	OR [95% CI] High Diet	p-value	OR [95% CI] hsCRP	p-value	OR [95% CI] Medium Diet	p-value	OR [95% CI] High Diet	p-value	OR [95% CI] hsCRP	p-value
PCS∼hPDI+hscrp	**1.119 [1.031; 1.214]**	**0.007**	**1.100 [1.006; 1.202]**	**0.036**	**0.984 [0.974; 0.993]**	**<0.001**	1.221 [0.991; 1.505]	0.061	1.158 [0.927; 1.448]	0.195	**0.972 [0.948; 0.994]**	**0.021**
PCS∼uPDI+hscrp	0.944 [0.869; 1.025]	0.168	**0.815 [0.747; 0.889]**	**<0.001**	**0.984 [0.975; 0.993]**	**<0.001**	0.883 [0.712; 1.094]	0.254	0.822 [0.664; 1.018]	0.072	**0.972 [0.948; 0.995]**	**0.022**
MCS∼hPDI+hsCRP	1.049 [0.967; 1.138]	0.253	**1.101 [1.007; 1.203]**	**0.034**	1.005 [0.996; 1.014]	0.258	0.984 [0.801; 1.209]	0.877	1.042 [0.836; 1.300]	0.712	1.001 [0.982; 1.021]	0.935
MCS∼uPDI+hsCRP	0.945 [0.870; 1.025]	0.173	**0.854 [0.783; 0.931]**	**<0.001**	1.005 [0.997; 1.014]	0.236	0.888 [0.718; 1.097]	0.271	0.908 [0.735; 1.121]	0.37	1.001 [0.982; 1.022]	0.913

n_older_=2,198, n_younger_=14,524, Adjusted for age, sex, BMI, physical activity, disease prevalence, income, smoking, alcohol intake, depression, and energy intake.

hPDI=healthful plant-based diet index, uPDI=unhealthful plant-based diet index, BMI=body mass index, Physical CS=physical component score, Mental CS=mental component score, hsCRP=high-sensitivity C-reactive protein, in bold=p<0.05.

### Sensitivity analysis

As the majority of the population is more likely to follow an overall plant-based diet including a mixture of healthful and unhealthful plant-based food, we carried out a sensitivity analysis with the overall plant-based diet index, to assess how intake of plant foods in general associates with quality of life. We observed a significant association between the highest tertile of PDI and physical quality of life in the whole sample (OR 1.05, p = 0.016), as well as with MCS (OR = 0.94, p = 0.03) (Supplementary Table 6).

Further, we replicated the analysis of an interactive effect between hsCRP and PDIs, as well as age-stratified analysis via a linear regression model with rank-inverse normal transformed component scores as the outcome. No interaction effect between hsCRP and PDIs was observed. Our results show overall similar results (Supplementary 7). Similarly, hsCRP was associated with physical and not mental quality of life in these models.

## Discussion

In this study, we find high physical and mental quality of life to be associated with high adherence to healthful plant-based diet. Similarly, low physical and mental quality of life is associated with high adherence to unhealthful plan-based diet. We note no differences between sexes. Further, although we observe significant interactions of PDIs and age, (Supplementary Table 8), effect sizes seem largely similar for adults below and above 60 years. The main difference lies in the narrower confidence intervals among the younger age group, potentially due to greater sample size. Overall, our findings suggest that the association between plant-based diet adherence and physical and mental quality of life does not significantly differ by age or sex. Inflammation, as measured by circulating hsCRP levels had no effect on the association between diet adherence and physical and mental quality of life, indicating independent effects.

Our findings on the association between plant-based diet adherence and quality of life, although cross-sectional, are consistent with earlier results^(7)^. Furthermore, our observation that hsCRP was significantly associated with physical, but not mental quality of life, is mainly in line with previous observations^(33,34)^. We extended previous research by assessing the association in men and women above 60 years of age. Further, we investigate state of inflammation as a potential mechanism explaining the association between plant-based diet adherence and quality of life. The interplay of diet, inflammation and quality of life was not assessed previously. By demonstrating that chronic inflammation levels and adherence to healthful plant-based diets are independently associated with physical and mental quality of life, we have elucidated some of the factors contributing to quality of life of older people and thereby likely to better survival into old age^(20)^. The independent nature of the effects of diet and inflammation also adds to disentangle the mechanisms through which diet affects health. Often the anti-inflammatory effects of plant-based diets is used as an explanation for health benefits associated with a plant-based diet. Since in our study the association of plant-based diets on quality of life was not dependent on levels of inflammation, this suggests other mechanisms apart from inflammation may be at play and should further be investigated.

The paradoxical relationship between ageing and quality of life, indicating that contrary to popular belief especially mental quality of life may be higher in older ages than in mid-life has been discussed in research^(35)^. This is confirmed in our sample: while older adults rated their physical quality of life lower than younger adults (51.7 vs 53.7), they showed higher mental quality of life (53.4 vs. 50.8). Further, older adults showed higher adherence to a healthful plant-based diet compared to younger adults (56.5 vs 53.5). Low variation in quality of life and diet scores among older adults may therefore explain why no significant effect was detected among this age group.

A potential explanation for the observed associations may therefore be reversed causation: those with higher quality of life, may have more energy and capacity to invest in a healthy diet. However, our analysis was adjusted for prevalence of depression and chronic diseases, suggesting that even taking into account these health issues, there is an effect of healthful plant-based diet on quality of life.

Another potential explanation for the observed association between a healthful plant-based diet and physical and mental quality of life may be explained by higher intakes in mono- and polyunsaturated fatty acids, plant proteins and fiber in those in the highest tertile of hPDI. These nutrients have previously been associated with lower prevalence of chronic diseases. In our analysis the association was observed despite adjusting for disease prevalence, suggesting other mechanisms may contribute to increased physical quality of life. Higher diet quality is further associated with higher antioxidant capacity^(36)^. Especially a high intake in plant proteins may be beneficial, as it contributes to maintaining muscle mass, while avoiding adverse health effects associated with high consumption of animal protein, such as an increased risk of insulin resistance and mortality^(37,38)^. At the same time, sugar intake was highest in highest tertile of uPDI, which could potentially have a negative effect on mood^(39)^. Since we found no interactive effect of hsCRP, an alternative explanation for the association between plant-based diets and quality of life may lie in the gut microbiome. Previously, a link between gut microbial features and quality of life and depression has been observed^(40,41)^.

Particularly a healthful plant-based diet has been associated with increased diversity and anti-inflammatory features of the gut microbiome^(42,43)^. Inflammation, a factor commonly assumed to play a role in the gut-brain axis, was however not a significant moderator in our analysis. An explanation may lie in our choice of inflammatory marker, as hsCRP is only one of many markers that can indicate inflammation. Previous research suggested that, for example glycoprotein acetyls may be a more sensitive marker for metabolic disease and gut microbiome function^(44)^. Apart from that, IL-6, as a marker of inflammation, has been found to be weakly but negatively associated with quality of life^(18)^. These markers were not available to us, but future studies may explore an interaction with novel markers of inflammation.

This study also has some important limitations. The study population was overall relatively healthy and had high quality of life scores compared to Dutch norms^(29)^. Also, levels of circulating hsCRP were only slightly elevated among the older age group compared to the younger age group (Table 1, Supplementary Table 2), indicating an overall good health among older adults in the sample. This may have led to low variation and difficulty detecting effects of elevated hsCRP levels. The mean age of our older age group of 65 years is also still relatively young. As critical aspects of plant-based diets, such as energy deficits, may only become a problem among very old adults, and also inflammation increases with age, repeating the analysis in this age group may have led to different results. Data on dietary intake and quality of life was self-reported, which could lead to bias due to underreporting or social desirability^(45)^. Secondly, we did not have data on vitamin D levels of participants available, although previous studies suggested an association of vitamin D levels with quality of life^(46,47)^. Further, we chose a logistic regression for our analysis, for which quality of life scores had to be binarized. The arbitrary cut-off at the median may be criticized, however a sensitivity analysis conducted by linear regression with normal-transformed component scores led to overall similar results (Supplementary Table 7). Lastly, this analysis was cross-sectional, meaning causality cannot be inferred. Accordingly, reverse causation could contribute to the findings. Previous research showed that stress and depressive symptoms can lead to less healthy food choices and have associated depressive symptoms to poorer diet quality^(48,49)^. However, meta-analysis of longitudinal studies suggests, that a high quality diet, may have a somewhat protective effect on depression risk^(50)^.

Strengths of our analysis include the large sample size from the population-representative Lifelines Cohort Study. Secondly, hsCRP is a widely used measure for inflammation, allowing for comparison with previously publications. The RAND-36 is a commonly used tool to assess quality of life and has also been validated in a Dutch population^(51)^. Similarly, the FFQ applied in the Lifelines Cohort Study has been found adequate for studying diet-disease associations and displayed good ranking abilities for most foods and nutrients^(23)^. The size and abundance of the Lifelines study allowed us to assess the interplay between plant-based diets, quality of life and inflammation in an age- and sex-diverse sample.

### Conclusion

In conclusion, we found a healthful plant-based diet to be associated with greater physical and mental quality of life in an age- and sex-diverse population, whereas the opposite association was found for adherence to an unhealthful plant-based diet. This effect was not dependent on circulating levels of hsCRP, while this inflammatory marker was independently associated with lower physical quality of life. A healthful plant-based diet may therefore contribute to sustain quality of life in both, younger and older adults. Future studies may focus on mechanisms that explain how plant-based diets may impact quality of life

## Supporting information

Schorr et al. supplementary material Schorr et al. supplementary material

## Footnotes

1
https://www.voedingscentrum.nl/nl/gezond-eten-met-de-schijf-van-vijf/wat-staat-in-de-schijf-van-vijf-en-wat-niet/olien-en-vetten.aspx

2
https://www.rand.org/health-care/surveys_tools/mos/36-item-short-form/scoring.html

## Supplementary material

The supplementary material for this article can be found at https://doi.org/10.1017/jns.2025.10023
