# Supplementary material for: A healthful plant-based diet is associated with higher health-related quality of life among older adults independent of circulating CRP: a cross-sectional analysis from the Lifelines Cohort Study
Source: J Nutr Sci. 2025 Aug 4;14:e54. doi: 10.1017/jns.2025.10023 (PMC12361675; doi:10.1017/jns.2025.10023)
Supplement: Schorr et al. supplementary material [file S2048679025100232sup001.docx]

Supplemenatry material **A healthful plant-based diet is associated with higher health-related quality of life among older adults independent of circulating CRP: a cross-sectional analysis from the Lifelines Cohort Study**

**Content:**

Suppl. Table 1 Categorization of food items and scoring of the PDI

Suppl. Table 2 Prevalence of age-related diseases in sample by age

Suppl. Table 3 Intakes of foodgroups per tertile of PDIs

Suppl. Table 4 Intakes of nutrients per tertile of PDIs

Suppl. Table 5 Effect sizes of covariates in different models

Suppl. Table 6 Sensitivity analysis: Association of overall plant-based diet index with physical and mental component score

Suppl. Table 7 Sensitivity analysis: Association of healthful and unhealthful plant-based diet with physical and mental component score by linear regression with rank-inverse normal transformation

Suppl. Material 1 Flowchart inclusion criteria

Suppl. Material 2 RAND-36 subdomains and examples

Suppl. Material 3 Overview over regression models

| Suppl. Table 1 Categorization of food items and scoring of the PDI | |
| --- | --- |
| ***Plant food groups*** |  |
| ***Healthy*** |  |
| **Vegetables** | Fresh vegetables, lettuce, potatoes without fat |
| **Fruits** | fresh fruit |
| **Nuts** | Nuts, peanut butter |
| **Legumes** | Legumes, soup with legumes, soy-based meat alternatives, soymilk |
| **Wholegrains** | Brown and wholegrain bread and breadrolls, wholegrain pasta and rice |
| **Vegetable Oils** | Vegetable oils used for cooking, oil-based salad dressings, margarines |
| **Tea & Coffee** | Tea and coffee including decaffeinated coffee |
| ***Less healthy*** |  |
| **Refined grains** | White bread, rice and pasta, crackers, chips, cruesli, raisin bread |
| **Fruit Juices** | Fruit juices and fruit drinks |
| **Potatoes** | Fried potatoes, fries, kroketten, potatoes prepared with butter/margarine |
| **Sweets & Desserts** | Chocolate sprinkles, syrup, sugar added to coffee, cakes/biscuits, tarts, candybars, chocolate |
| **Sugar sweetened beverages** | Soda |
| ***Animal food groups*** |  |
| **Animal fats** | Butter, lard |
| **Eggs** | Eggs |
| **Dairy** | Milk, including milk in coffee, cheese, ice cream, yogurt |
| **Fish** | Dark meat fish, other fish, seafood, fried fish, salads with fish |
| **Meat & Meatproducts** | Chicken, pork, beef, bacon, sausages, burgers, offal |
| **Miscellaneous animal-based foods** | Pizza, salades, kroket |

| Suppl. Table 2 Prevalence of age-related diseases in sample by age | | |
| --- | --- | --- |
| Disease prevalence | younger | older |
| n | 35,447 | 6,528 |
| Heartattack n(%) | 149 (0.4%) | 227 (3.5%) |
| Stroke n(%) | 148 (0.4%) | 111 (1.7%) |
| Hypertension n(%) | 6371 (18.0%) | 2602 (39.9%) |
| T2D n(%) | 305 (0.9%) | 369 (5.7%) |
| Arthritis n(%) | 552 (1.6%) | 290 (0.4%) |
| Chestpain n(%) | 8631 (24.4%) | 1650 (25.3%) |
| Cancer n(%) | 1270 (3.6%) | 793 (12.1%) |

Suppl. Table 3 Intakes of foodgroups per tertile of healthful and unhealthful plant-based diet index

|  | older | | | | | | younger | | | | | |
| --- | --- | --- | --- | --- | --- | --- | --- | --- | --- | --- | --- | --- |
|  | hPDI | | | uPDI | | | hPDI | | | uPDI | | |
| **Food Group** | **low** | **medium** | **high** | **low** | **medium** | **high** | **low** | **medium** | **high** | **low** | **medium** | **high** |
|  | mean (SD) | mean (SD) | mean (SD) | mean (SD) | mean (SD) | mean (SD) | mean (SD) | mean (SD) | mean (SD) | mean (SD) | mean (SD) | mean (SD) |
| Animal fats | 0.9(1.68) | 0.6(1.49) | 0.5(1.33) | 0.9(1.65) | 0.7(1.55) | 0.5(1.37) | 1.3(1.84) | 0.9(1.69) | 0.5(1.39) | 0.8(1.61) | 0.8(1.65) | 0.8(1.65) |
| Dairy | 17.8(4.99) | 17.8(4.99) | 17.7(5.12) | 18.6(5.00) | 17.8(4.97) | 16.9(4.97) | 18.6(4.49) | 18.6(4.63) | 18.3(4.76) | 18.9(4.60) | 18.0(4.71) | 17.7(4.67) |
| Eggs | 3.5(1.56) | 3.3(1.52) | 3.1(1.52) | 3.7(1.57) | 3.3(1.46) | 2.9(1.47) | 3.9(1.49) | 3.7(1.51) | 3.4(1.51) | 3.9(1.50) | 3.3(1.44) | 3.1(1.57) |
| Fish | 3.4(2.21) | 3.3(2.24) | 3.1(2.33) | 4.2(2.15) | 3.3(2.16) | 2.4(2.11) | 4.2(2.08) | 4.0(2.16) | 3.7(2.26) | 4.4(2.07) | 3.5(2.19) | 2.8(2.23) |
| Fruit | 8.5(4.28) | 10.4(4.51) | 12.2(4.55) | 12.1(4.35) | 10.3(4.53) | 8.3(4.39) | 10.3(4.56) | 12.3(4.46) | 13.8(4.21) | 13.4(4.14) | 11.9(4.64) | 10.0(5.06) |
| Fruitjuice | 7.1(5.24) | 5.6(5.12) | 4.3(4.93) | 4.2(4.59) | 5.8(5.06) | 7.3(5.52) | 6.3(5.02) | 4.8(4.63) | 3.4(4.40) | 3.6(4.32) | 5.3(5.01) | 6.5(5.04) |
| Leegumes | 3.0(2.94) | 4.1(3.37) | 5.2(3.74) | 5.0(3.57) | 4.0(3.39) | 3.0(3.07) | 3.4(2.86) | 4.2(3.29) | 5.3(3.53) | 5.0(3.35) | 4.1(3.45) | 3.5(3.09) |
| Meat | 8.9(2.12) | 8.7(2.38) | 8.1(2.83) | 8.7(2.56) | 8.6(2.43) | 8.4(2.35) | 8.6(2.02) | 8.3(2.25) | 7.8(2.60) | 8.3(2.39) | 8.0(2.39) | 7.8(2.38) |
| Miscelleneous animal products | 5.5(1.97) | 4.7(2.06) | 4.0(2.13) | 4.5(2.12) | 4.8(2.10) | 5.0(2.16) | 3.8(2.14) | 3.1(2.04) | 2.6(1.94) | 3.1(2.06) | 3.0(2.08) | 3.0(2.07) |
| Nuts | 2.7(1.64) | 3.2(1.78) | 3.9(2.00) | 3.6(1.84) | 3.2(1.82) | 2.8(1.84) | 2.3(1.56) | 2.8(1.71) | 3.5(1.93) | 3.3(1.82) | 2.8(1.82) | 2.4(1.75) |
| Potatoes | 5.8(2.41) | 5.1(2.51) | 4.4(2.57) | 4.3(2.40) | 5.2(2.46) | 6.0(2.53) | 5.4(3.04) | 4.4(3.07) | 3.6(3.03) | 3.6(2.82) | 4.8(3.19) | 6.0(3.41) |
| Refined Grains | 10.0(2.52) | 9.7(2.67) | 9.2(2.90) | 8.9(2.66) | 9.8(2.59) | 10.4(2.63) | 8.7(2.52) | 8.3(2.53) | 7.9(2.79) | 7.7(2.58) | 8.6(2.53) | 9.4(2.82) |
| Soda | 8.5(6.51) | 5.9(5.97) | 3.8(5.13) | 3.1(4.31) | 5.9(5.61) | 9.7(6.67) | 4.9(5.16) | 3.2(4.48) | 2.0(3.42) | 1.8(3.17) | 4.1(4.83) | 6.0(5.54) |
| Sweets & Desserts | 9.3(2.47) | 9.1(2.58) | 8.9(2.66) | 8.0(2.34) | 9.1(2.38) | 10.2(2.49) | 9.1(2.41) | 8.9(2.45) | 8.8(2.60) | 8.3(2.36) | 9.5(2.46) | 10.3(2.36) |
| Tea & Coffee | 22.7(6.50) | 25.2(5.96) | 27.3(5.59) | 27.1(5.38) | 25.0(5.94) | 22.4(6.73) | 23.9(5.76) | 25.7(5.40) | 27.6(5.19) | 27.0(5.26) | 25.4(5.66) | 24.1(6.04) |
| Vegetable oil | 4.1(1.68) | 4.9(1.82) | 5.4(1.92) | 4.8(1.83) | 4.8(1.86) | 4.6(1.91) | 4.1(1.72) | 4.6(1.75) | 5.3(1.82) | 4.9(1.78) | 4.7(1.86) | 4.6(2.04) |
| Vegetables | 12.9(2.98) | 14.4(3.12) | 15.9(3.27) | 15.7(3.15) | 14.2(3.11) | 12.8(3.12) | 12.9(3.10) | 14.2(3.08) | 15.7(3.29) | 15.4(3.11) | 13.8(3.33) | 12.6(3.27) |
| Wholegrains | 8.6(3.07) | 10.2(2.98) | 11.7(3.00) | 10.8(3.05) | 10.2(3.16) | 9.2(3.35) | 8.0(3.00) | 9.3(2.79) | 10.8(2.87) | 10.2(2.91) | 9.3(3.00) | 8.4(3.48) |

Suppl. Table 4 Nutrient intakes in older adults (>=60years) per tertile of healthful (hPDI) and unhealthful (uPDI) plant-based diet index

|  | hPDI | | | **uPDI** | | |
| --- | --- | --- | --- | --- | --- | --- |
| **intake (SD)** | **low** | **medium** | **high** | **low** | **medium** | **high** |
| Nutrients |  |  |  |  |  |  |
| Alcohol [g/d] | 8.6(9.53) | 8.4(9.00) | 8.5(9.44) | 9.1(9.32) | 7.9(9.24) | 7.2(9.29) |
| Energy [kcal/d] | 1,007.7(320.94) | 1,037.7(328.33) | 1,097.4(348.72) | 1,091.8(337.35) | 1,020.8(328.34) | 990.8(344.34) |
| DHA [g/d] | 0.1(0.11) | 0.1(0.11) | 0.1(0.12) | 0.1(0.12) | 0.1(0.11) | 0.1(0.09) |
| Protein [g/d] | 73.6(15.82) | 75.1(16.21) | 78.9(17.28) | 79.2(16.42) | 73.7(16.36) | 70.5(16.61) |
| Animal protein [g/d] | 47.3(12.30) | 45.8(12.32) | 44.2(13.07) | 47.3(12.71) | 43.5(12.13) | 41.5(12.49) |
| Plant protein [g/d] | 26.4(6.78) | 29.4(7.64) | 34.7(9.29) | 32.1(8.95) | 30.3(8.88) | 29.1(8.58) |
| EPA [g/d] | 0.1(0.08) | 0.1(0.08) | 0.1(0.08) | 0.1(0.09) | 0.1(0.08) | 0.1(0.07) |
| Fat [g/d] | 80.9(27.28) | 79.9(27.45) | 85.3(29.67) | 81.6(27.60) | 82.9(29.48) | 86.4(30.28) |
| Folate equivalents | 238.7(67.56) | 265.5(77.67) | 301.4(92.30) | 292.7(87.00) | 260.4(80.02) | 235.3(77.05) |
| Carbohydrates [g/d] | 204.9(53.80) | 208.7(53.78) | 222.3(57.61) | 207.0(52.54) | 219.8(58.08) | 232.4(61.00) |
| Monosaccharides [g/d] | 99.5(34.78) | 97.8(32.42) | 98.1(33.55) | 91.7(28.73) | 104.2(34.78) | 114.8(41.01) |
| MUFA [g/d] | 27.6(9.65) | 27.5(10.07) | 29.4(11.11) | 28.3(10.29) | 28.3(10.76) | 29.2(10.88) |
| PUFA[g/d] | 15.6(6.96) | 16.5(7.44) | 19.5(9.06) | 17.6(8.00) | 17.6(8.55) | 18.0(8.99) |
| SFA [g/d] | 30.6(10.96) | 29.0(10.31) | 29.3(10.19) | 28.7(9.94) | 29.9(10.79) | 32.0(11.20) |
| Fibre [g/d] | 19.3(4.83) | 21.9(5.21) | 25.8(6.29) | 24.1(6.17) | 22.2(6.00) | 20.8(6.17) |
| Vitamin B12 [mcg/d] | 5.0(2.50) | 4.6(2.32) | 4.3(2.17) | 4.8(2.41) | 4.2(2.12) | 3.9(2.04) |

Suppl. Table 5 Effect sizes of covariates in different models

|  | hPDI | | | | | | | | uPDI | | | | | | | |
| --- | --- | --- | --- | --- | --- | --- | --- | --- | --- | --- | --- | --- | --- | --- | --- | --- |
|  | PCS | | | | MCS | | | | PCS | | | | MCS | | | |
| Older adults | OR | LCI  95% | UCI  95% | p | OR | LCI  95% | UCI  95% | p | OR | LCI  95% | UCI  95% | p | OR | LCI  95% | UCI  95% | p |
| Model 1 |  |  |  |  |  |  |  |  |  |  |  |  |  |  |  |  |
| hPDI (medium) | 1.08 | 0.95 | 1.23 | 0.22 | 1.03 | 0.91 | 1.16 | 0.67 | 0.90 | 0.79 | 1.02 | 0.10 | 0.92 | 0.82 | 1.04 | 0.19 |
| hPDI (high) | 1.14 | 1.00 | 1.31 | 0.05 | 1.13 | 1.00 | 1.28 | 0.06 | 0.84 | 0.74 | 0.96 | 0.01 | 0.89 | 0.78 | 1.01 | 0.07 |
| Age | 0.97 | 0.96 | 0.98 | 0.00 | 1.02 | 1.01 | 1.03 | 0.00 | 0.97 | 0.96 | 0.98 | 0.00 | 1.02 | 1.01 | 1.03 | 0.00 |
| Sex (male) | 1.38 | 1.22 | 1.56 | 0.00 | 1.79 | 1.60 | 2.01 | 0.00 | 1.38 | 1.22 | 1.56 | 0.00 | 1.78 | 1.59 | 2.00 | 0.00 |
| Physical Activity (h/wk) | 1.02 | 1.01 | 1.02 | 0.00 | 1.00 | 1.00 | 1.01 | 0.29 | 1.02 | 1.01 | 1.02 | 0.00 | 1.00 | 1.00 | 1.01 | 0.30 |
| Bmi | 0.92 | 0.91 | 0.94 | 0.00 | 1.04 | 1.03 | 1.06 | 0.00 | 0.92 | 0.90 | 0.93 | 0.00 | 1.04 | 1.02 | 1.05 | 0.00 |
| Income | 1.12 | 1.03 | 1.21 | 0.01 | 1.21 | 1.12 | 1.32 | 0.00 | 1.11 | 1.02 | 1.21 | 0.02 | 1.21 | 1.12 | 1.31 | 0.00 |
| Nr of diseases | 0.65 | 0.61 | 0.70 | 0.00 | 0.85 | 0.80 | 0.90 | 0.00 | 0.65 | 0.61 | 0.70 | 0.00 | 0.85 | 0.80 | 0.90 | 0.00 |
| Alcohol intake (g/d) | 1.01 | 1.00 | 1.01 | 0.07 | 1.00 | 0.99 | 1.01 | 0.88 | 1.00 | 1.00 | 1.01 | 0.11 | 1.00 | 0.99 | 1.01 | 0.98 |
| Smoker | 1.29 | 1.06 | 1.57 | 0.01 | 1.21 | 1.01 | 1.46 | 0.04 | 1.28 | 1.06 | 1.57 | 0.01 | 1.21 | 1.00 | 1.46 | 0.05 |
| Depression (no) | 1.12 | 0.91 | 1.37 | 0.28 | 3.63 | 2.92 | 4.55 | 0.00 | 1.12 | 0.91 | 1.37 | 0.29 | 3.61 | 2.91 | 4.53 | 0.00 |
| Energy intake (kcal/d) | 1.00 | 1.00 | 1.00 | 0.00 | 1.00 | 1.00 | 1.00 | 0.00 | 1.00 | 1.00 | 1.00 | 0.00 | 1.00 | 1.00 | 1.00 | 0.00 |
| Model 2 |  |  |  |  |  |  |  |  |  |  |  |  |  |  |  |  |
| hPDI (medium) | 1.22 | 0.99 | 1.50 | 0.06 | 0.98 | 0.80 | 1.21 | 0.88 | 0.88 | 0.71 | 1.09 | 0.25 | 0.89 | 0.72 | 1.10 | 0.27 |
| hPDI (high) | 1.16 | 0.93 | 1.45 | 0.20 | 1.04 | 0.84 | 1.30 | 0.71 | 0.82 | 0.66 | 1.02 | 0.07 | 0.91 | 0.74 | 1.12 | 0.37 |
| hsCRP (mg/L) | 0.97 | 0.95 | 0.99 | 0.02 | 1.00 | 0.98 | 1.02 | 0.93 | 0.97 | 0.95 | 0.99 | 0.02 | 1.00 | 0.98 | 1.02 | 0.91 |
| Age | 1.46 | 1.20 | 1.79 | 0.00 | 1.90 | 1.56 | 2.32 | 0.00 | 1.46 | 1.19 | 1.78 | 0.00 | 1.91 | 1.57 | 2.33 | 0.00 |
| Sex (male) | 0.97 | 0.95 | 0.99 | 0.00 | 1.02 | 1.01 | 1.04 | 0.01 | 0.97 | 0.95 | 0.99 | 0.00 | 1.02 | 1.01 | 1.04 | 0.01 |
| Physical Activity (h/wk) | 1.02 | 1.00 | 1.03 | 0.01 | 1.00 | 0.99 | 1.01 | 0.44 | 1.02 | 1.00 | 1.03 | 0.01 | 1.00 | 0.98 | 1.01 | 0.41 |
| Bmi | 0.94 | 0.92 | 0.96 | 0.00 | 1.03 | 1.01 | 1.06 | 0.01 | 0.94 | 0.91 | 0.96 | 0.00 | 1.03 | 1.01 | 1.06 | 0.02 |
| Income | 1.23 | 1.07 | 1.42 | 0.00 | 1.17 | 1.02 | 1.35 | 0.03 | 1.22 | 1.06 | 1.41 | 0.01 | 1.16 | 1.01 | 1.34 | 0.04 |
| Nr of diseases | 0.70 | 0.63 | 0.78 | 0.00 | 0.92 | 0.83 | 1.01 | 0.08 | 0.70 | 0.63 | 0.78 | 0.00 | 0.91 | 0.83 | 1.01 | 0.07 |
| Alcohol intake (g/d) | 1.01 | 1.00 | 1.02 | 0.02 | 1.00 | 0.99 | 1.01 | 0.98 | 1.01 | 1.00 | 1.02 | 0.03 | 1.00 | 0.99 | 1.01 | 0.94 |
| Smoker | 1.06 | 0.76 | 1.46 | 0.74 | 1.29 | 0.93 | 1.79 | 0.12 | 1.06 | 0.77 | 1.47 | 0.71 | 1.28 | 0.93 | 1.78 | 0.13 |
| Depression (no) | 1.34 | 0.96 | 1.87 | 0.08 | 3.60 | 2.50 | 5.29 | 0.00 | 1.35 | 0.97 | 1.88 | 0.08 | 3.58 | 2.49 | 5.26 | 0.00 |
| Energy intake (kcal/d) | 1.00 | 1.00 | 1.00 | 0.08 | 1.00 | 1.00 | 1.00 | 0.02 | 1.00 | 1.00 | 1.00 | 0.12 | 1.00 | 1.00 | 1.00 | 0.02 |
| **Younger adults** |  |  |  |  |  |  |  |  |  |  |  |  |  |  |  |  |
| **Model 1** |  |  |  |  |  |  |  |  |  |  |  |  |  |  |  |  |
| hPDI (medium) | 1.12 | 1.06 | 1.18 | 0.00 | 1.06 | 1.00 | 1.11 | 0.04 | 0.92 | 0.87 | 0.97 | 0.00 | 0.90 | 0.85 | 0.95 | 0.00 |
| hPDI (high) | 1.13 | 1.07 | 1.20 | 0.00 | 1.10 | 1.04 | 1.16 | 0.00 | 0.83 | 0.78 | 0.88 | 0.00 | 0.85 | 0.81 | 0.90 | 0.00 |
| Age | 0.98 | 0.98 | 0.98 | 0.00 | 1.02 | 1.01 | 1.02 | 0.00 | 0.98 | 0.97 | 0.98 | 0.00 | 1.02 | 1.01 | 1.02 | 0.00 |
| Sex (male) | 1.29 | 1.22 | 1.36 | 0.00 | 1.60 | 1.51 | 1.68 | 0.00 | 1.27 | 1.21 | 1.34 | 0.00 | 1.59 | 1.50 | 1.67 | 0.00 |
| Physical Activity (h/wk) | 1.00 | 1.00 | 1.00 | 0.01 | 1.00 | 1.00 | 1.01 | 0.00 | 1.00 | 1.00 | 1.00 | 0.01 | 1.00 | 1.00 | 1.01 | 0.00 |
| Bmi | 0.93 | 0.93 | 0.94 | 0.00 | 1.02 | 1.02 | 1.03 | 0.00 | 0.93 | 0.92 | 0.94 | 0.00 | 1.02 | 1.02 | 1.03 | 0.00 |
| Income | 1.14 | 1.11 | 1.18 | 0.00 | 1.21 | 1.17 | 1.25 | 0.00 | 1.14 | 1.10 | 1.17 | 0.00 | 1.20 | 1.17 | 1.25 | 0.00 |
| Nr of diseases | 0.62 | 0.60 | 0.64 | 0.00 | 0.72 | 0.70 | 0.74 | 0.00 | 0.62 | 0.60 | 0.64 | 0.00 | 0.72 | 0.70 | 0.74 | 0.00 |
| Alcohol intake (g/d) | 1.00 | 1.00 | 1.01 | 0.00 | 1.00 | 1.00 | 1.00 | 0.63 | 1.00 | 1.00 | 1.01 | 0.00 | 1.00 | 1.00 | 1.00 | 0.98 |
| Smoker | 1.24 | 1.17 | 1.32 | 0.00 | 1.11 | 1.04 | 1.17 | 0.00 | 1.24 | 1.17 | 1.32 | 0.00 | 1.10 | 1.04 | 1.17 | 0.00 |
| Depression (no) | 1.25 | 1.16 | 1.35 | 0.00 | 3.51 | 3.22 | 3.82 | 0.00 | 1.25 | 1.16 | 1.35 | 0.00 | 3.50 | 3.21 | 3.81 | 0.00 |
| Energy intake (kcal/d) | 1.00 | 1.00 | 1.00 | 0.00 | 1.00 | 1.00 | 1.00 | 0.00 | 1.00 | 1.00 | 1.00 | 0.05 | 1.00 | 1.00 | 1.00 | 0.00 |
| Model 2 |  |  |  |  |  |  |  |  |  |  |  |  |  |  |  |  |
| hPDI (medium) | 1.12 | 1.03 | 1.21 | 0.01 | 1.05 | 0.80 | 1.21 | 0.25 | 0.94 | 0.87 | 1.02 | 0.17 | 0.94 | 0.87 | 1.03 | 0.17 |
| hPDI (high) | 1.10 | 1.01 | 1.20 | 0.04 | 1.10 | 0.84 | 1.30 | 0.03 | 0.82 | 0.75 | 0.89 | 0.00 | 0.85 | 0.78 | 0.93 | 0.00 |
| hsCRP (mg/L) | 0.98 | 0.97 | 0.99 | 0.00 | 1.01 | 0.98 | 1.02 | 0.26 | 0.98 | 0.97 | 0.99 | 0.00 | 1.01 | 1.00 | 1.01 | 0.24 |
| Age | 1.19 | 1.10 | 1.30 | 0.00 | 1.58 | 1.56 | 2.32 | 0.00 | 1.18 | 1.09 | 1.29 | 0.00 | 1.57 | 1.44 | 1.70 | 0.00 |
| Sex (male) | 0.98 | 0.97 | 0.98 | 0.00 | 1.02 | 1.01 | 1.04 | 0.00 | 0.98 | 0.97 | 0.98 | 0.00 | 1.02 | 1.01 | 1.02 | 0.00 |
| Physical Activity (h/wk) | 1.00 | 1.00 | 1.00 | 0.91 | 1.01 | 0.99 | 1.01 | 0.00 | 1.00 | 1.00 | 1.00 | 0.85 | 1.01 | 1.00 | 1.01 | 0.00 |
| Bmi | 0.94 | 0.93 | 0.95 | 0.00 | 1.02 | 1.01 | 1.06 | 0.00 | 0.94 | 0.93 | 0.95 | 0.00 | 1.02 | 1.01 | 1.03 | 0.00 |
| Income | 1.13 | 1.07 | 1.19 | 0.00 | 1.21 | 1.02 | 1.35 | 0.00 | 1.12 | 1.06 | 1.18 | 0.00 | 1.20 | 1.14 | 1.26 | 0.00 |
| Nr of diseases | 0.61 | 0.58 | 0.64 | 0.00 | 0.70 | 0.83 | 1.01 | 0.00 | 0.61 | 0.58 | 0.64 | 0.00 | 0.70 | 0.66 | 0.73 | 0.00 |
| Alcohol intake (g/d) | 1.00 | 1.00 | 1.01 | 0.02 | 1.00 | 0.99 | 1.01 | 0.50 | 1.00 | 1.00 | 1.01 | 0.06 | 1.00 | 1.00 | 1.00 | 0.71 |
| Smoker | 1.25 | 1.14 | 1.36 | 0.00 | 1.08 | 0.93 | 1.79 | 0.08 | 1.24 | 1.14 | 1.36 | 0.00 | 1.08 | 0.99 | 1.19 | 0.08 |
| Depression (no) | 1.28 | 1.14 | 1.43 | 0.00 | 3.51 | 2.50 | 5.29 | 0.00 | 1.27 | 1.13 | 1.42 | 0.00 | 3.49 | 3.07 | 3.98 | 0.00 |
| Energy intake (kcal/d) | 1.00 | 1.00 | 1.00 | 0.51 | 1.00 | 1.00 | 1.00 | 0.00 | 1.00 | 1.00 | 1.00 | 0.98 | 1.00 | 1.00 | 1.00 | 0.00 |

LCI95%: lower 95% confidence interval, UCI95%: upper 95% confidence interval

Suppl. Table 6 Sensitivity analysis: Association of overall plant-based diet index with physical and mental component score

| Predictor | OR | LCI 95% | UCI 95% | p |
| --- | --- | --- | --- | --- |
| PCS |  |  |  |  |
| PDI (medium) | 1.02 | 0.97 | 1.07 | 0.54 |
| PDI (high) | 1.05 | 1.00 | 1.11 | 0.05 |
| Age | 0.98 | 0.98 | 0.98 | 0.00 |
| Sex (male) | 1.30 | 1.24 | 1.37 | 0.00 |
| Physical Activity (h/wk) | 1.00 | 1.00 | 1.01 | 0.00 |
| Bmi | 0.93 | 0.93 | 0.94 | 0.00 |
| Income | 1.16 | 1.12 | 1.19 | 0.00 |
| Nr of diseases | 0.62 | 0.60 | 0.64 | 0.00 |
| Alcohol intake (g/d) | 1.01 | 1.00 | 1.01 | 0.00 |
| Smoker | 1.26 | 1.19 | 1.33 | 0.00 |
| Depression (no) | 1.28 | 1.19 | 1.37 | 0.00 |
| Energy intake (kcal/d) | 1.00 | 1.00 | 1.00 | 0.00 |
| MCS |  |  |  |  |
| PDI (medium) | 0.96 | 0.92 | 1.01 | 0.10 |
| PDI (high) | 0.94 | 0.90 | 0.99 | 0.03 |
| Age | 1.02 | 1.02 | 1.02 | 0.00 |
| Sex (male) | 1.65 | 1.57 | 1.73 | 0.00 |
| Physical Activity (h/wk) | 1.00 | 1.00 | 1.01 | 0.00 |
| Bmi | 1.02 | 1.01 | 1.03 | 0.00 |
| Income | 1.20 | 1.16 | 1.23 | 0.00 |
| Nr of diseases | 0.76 | 0.74 | 0.78 | 0.00 |
| Alcohol intake (g/d) | 1.00 | 1.00 | 1.00 | 0.84 |
| Smoker | 1.14 | 1.08 | 1.21 | 0.00 |
| Depression (no) | 3.59 | 3.32 | 3.90 | 0.00 |
| Energy intake (kcal/d) | 1.00 | 1.00 | 1.00 | 0.00 |

LCI95%: lower 95% confidence interval, UCI95%: upper 95% confidence interval

Suppl. Table 7 Association between healthful and unhealthful plant-based diet with physical and mental component score

|  | PCS | | | | MCS | | | |
| --- | --- | --- | --- | --- | --- | --- | --- | --- |
| Model 1 | OR | LCI 95% | UCI 95% | p | OR | LCI 95% | UCI 95% | p |
| Older adults | |  |  |  |  |  |  |  |
| hPDI | 0.03 | 1.00 | 1.05 | 0.03 | 0.02 | 0.99 | 1.04 | 0.14 |
| uPDI | -0.03 | 0.95 | 0.99 | 0.01 | -0.03 | 0.95 | 0.99 | 0.01 |
| Younger radults | |  |  |  |  |  |  |  |
| hPDI | 0.03 | 1.02 | 1.04 | 0.00 | 0.02 | 1.01 | 1.03 | 0.00 |
| uPDI | -0.03 | 0.96 | 0.98 | 0.00 | -0.03 | 0.96 | 0.98 | 0.00 |
| Model 2 |  |  |  |  |  |  |  |  |
| Older adults | |  |  |  |  |  |  |  |
| hPDI | 0.03 | 0.99 | 1.07 | 0.19 | 0.01 | 0.97 | 1.05 | 0.61 |
| uPDI | -0.02 | 0.94 | 1.02 | 0.33 | -0.01 | 0.95 | 1.03 | 0.55 |
| Younger radults | |  |  |  |  |  |  |  |
| hPDI | 0.03 | 1.01 | 1.05 | 0.00 | 0.02 | 1.01 | 1.04 | 0.01 |
| uPDI | -0.03 | 0.96 | 0.99 | 0.00 | -0.03 | 0.96 | 0.99 | 0.00 |

Model 1:adjusted by age, sex, bmi, Physical activity, income, energy intake, nr of diseases, depression

Model 2: adjusted by age, sex, bmi, Physical activity, income, energy intake, nr of diseases, depression, hsCRP

LCI95%: lower 95% confidence interval, UCI95%: upper 95% confidence interval

Lifelines Cohort Study

n=167,729

Final sample

n=41,975

Excluding participants with missing/unreliable dietary, hQOL or covariate data
n=125,244

Younger adults

n=14,524

Older adults

n=2,198

Exclude participants with missing data for hsCRP
n=25,253

Younger adults

n=35,447

Older adults

n=6,528

**Suppl. Figure 1**: Flowchart detailing inclusion process

**Suppl. Material 1: Subdomains and example questions in the RAND-36 Questionnaire**

Physical Functioning: if and to what extent participants are limited in performing daily activities, such as vigorous and moderate activity, carrying groceries, climbing stairs, walking etc.

Role limitations due to physical health: if participants had problems at work or other daily activities due to their physical health, such as cutting down on work time, accomplished less than they would like or were limited in the type of work/activity they could perform

Role limitations due to emotional problems: if participants had problems at work or other daily activities due to their emotional problems, such as cutting down on work time, accomplished less than they would like or couldn’t do work or other activities as usual

Energy/fatigue: how often participants have felt in the past 4 weeks full of energy, worn out or tired

Emotional well-being: how often participants have felt in the past 4 weeks nervous, down, peaceful or happy

Social functioning: to what extent has physical or emotional health interfered with normal social activities

Pain: how much pain participants had in the past 4 week and to what extent this interfered with their normal work

General health: how participants rate their own health

**Suppl. Material 2: Overview over regression models**

Model 1: assess associations between physical (PCS) and mental component scores (MCS) with PDIs in whole sample

1 PCS/MCS~hPDI/uPDI+covariates

Model 2: assess interaction with sex and age

2.1 PCS/MCS~hPDI/uPDI*sex+covariates

2.2 PCS/MCS~hPDI/uPDI*age+covariates

2.2.1 PCS/MCS~hPDI+covariates in older (above 60 years) and younger adults (below 60 years)

Model 3: assess interaction or independent effect of PDIs with hsCRP in older and younger adults

3.1 PCS/MCS~hPDI/uPDI*hsCRP+covariates in younger and older adults

3.2 PCS/MCS~hPDI/uPDI+hsCRP+covariates in younger and older adults

Sensitivity Analysis with overall PDI (in 4 groups stratified by age above/below 60 years):

1. Mental Component Score ~ overall PDI + covariates
2. Physical Component Score ~ overall PDI + covariates

Replication of steps 1-3 with linear regression and continuous component scores
